# Are We Underestimating Benthic Cyanotoxins? Extensive Sampling Results from Spain

**DOI:** 10.3390/toxins9120385

**Published:** 2017-11-28

**Authors:** Enrique A. Cantoral Uriza, Antonia D. Asencio, Marina Aboal

**Affiliations:** 1 Unidad Multidisciplinaria de Docencia e Investigación (UMDI), Facultad de Ciencias, Universidad Nacional Autónoma de México, Campus Juriquilla, C.P. Querétaro 76230, Mexico; cantoral@ciencias.unam.mx; 2 Departamento de Biología Aplicada (Botánica), Facultad de Ciencias Experimentales, Universidad Miguel Hernández, Campus de Elche, E-03202 Alicante, Spain; aasencio@umh.es; 3 Laboratorio de Algología, Departamento de Biología Vegetal, Facultad de Biología, Universidad de Murcia, Campus de Espinardo, E-30100 Murcia, Spain

**Keywords:** Anatoxin-a, aquatic and aerophytic habitats, cyanobacteria, microcystins, MC-LF, MC-LR, MC-LY, MC-RR, MC-YR, Spain

## Abstract

Microcystins (MCs) are potent hepatotoxins, and their presence in water bodies poses a threat to wildlife and human populations. Most of the available information refers to plankton, and much less is known about microcystins in other habitats. To broaden our understanding of the presence and environmental distribution of this group of toxins, we conducted extensive sampling throughout Spain, under a range of conditions and in distinct aquatic and terrestrial habitats. More than half of the tested strains were toxic; concentrations of the hepatotoxin were low compared with planktic communities, and the number of toxic variants identified in each sample of the Spanish strains ranged from 1–3. The presence of microcystins LF and LY (MC-LF and MC-LY) in the tested samples was significant, and ranged from 21.4% to 100% of the total microcystins per strain. These strains were only detected in cyanobacteria Oscillatoriales and Nostocales. We can report, for the first time, seven new species of microcystin producers in high mountain rivers and chasmoendolithic communities. This is the first report of these species in *Geitlerinema* and the confirmation of Anatoxin-a in *Phormidium uncinatum*. Our findings show that microcystins are widespread in all habitat types, including both aerophytic and endolithic peat bogs and that it is necessary to identify all the variants of microcystins in aquatic bodies as the commonest toxins sometimes represent a very low proportion of the total.

## 1. Introduction

Benthic cyanobacteria that produce microcystins (MCs) were first detected and characterised in communities in alpine lakes [1]. Since then, their presence in benthic communities in Spain [2,3], California [4], Egypt [5], Morocco [6,7], New Zealand [8], and other countries [9] has been reported. More recently, the presence of benthic cyanobacteria has been demonstrated in 30% of fordable rivers in California, and the number of potentially toxic genera has increased [10].

Foliaceus lichens of the genera *Nephroma*, *Sticta*, *Lobaria,* and *Peltigera* produce microcystins, including some new variants [11,12]. All these lichens contain the cyanobacteria *Nostoc* as a phycosymbiont. Some *Nostocs* found outside of lichens can also produce MCs [13], and their capacity to do so has been connected to stress conditions. However, no toxins have been detected in *Nostoc commune* var. *flagelliforme* Bornet and Flahault, which is common in desert regions, and is frequently consumed as food in countries such as China [14].

Van Apeldoorn et al. [15] reported a total of 40 toxic genera of benthic cyanobacteria, but the number has increased since that report [16]. However, most efforts to study these bacteria have focused on studying planktic communities, lakes, and reservoirs.

Aside from their toxicity to humans, the actual role of microcystins in the environment is not completely understood, in either planktic or benthic algal communities [17]. There is a large body of literature about the toxic effects of microcystins on angiosperms, fungi, bacteria, animals, and algae, but much more has to be done in order to elucidate this complex issue. These compounds interfere with biological processes that are necessary for the survival of organisms. They are potent inhibitors of certain essential protein phosphatases (PP1 and PP2) for all eukaryotes [18,19], and affect microalgae growth [20]. However, the promotion or suppression of photosynthesis has also been observed in several types of benthic algae, including diatoms [21], which are the food preferred by many herbivores. Microcystins may also inhibit the proteases employed in the digestion of heterotrophs [22], and evidence suggests that their presence in water may cause histological degeneration in macroinvertebrates, although the survival ratios are relatively high below 5 ppb of MC-LR and MC-LW [23]. Toxins may also accumulate in fish tissue, and pathological effects have been studied extensively [24,25].

**Figure 1:**
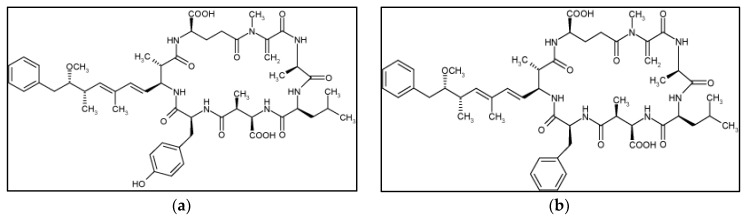
Structure of microcystins (**a**) LF (MC-LF) and (**b**) LY (MC-LY).

In recent years, the frequency of what have, until now, been considered rare variants of microcystins (Figure 1) has been confirmed in several countries, using a variety of methods [26,27,28,29,30]. Molecular tools have advanced considerably in the last few years, but quantitative methods are still not adequate for detecting toxicity. In most cases, the relationship between gene expression and toxin production is not yet sufficiently understood [31]. Most environmental agencies recommend using different complementary methodologies to detect and quantify microcystins [18]. 

Benthic toxicity was initially related to Oscillatoriales and Anatoxin-a [32,33]. However, it seems that these toxins are not as prevalent as originally thought, at least in some countries or geographical areas [34]. Some species of cyanobacteria may produce both microcystins and Anatoxin-a at the same time [35,36], although most studies focus on only one type of toxin at a time. Anatoxin-a (ANT-a) is a neurotoxic alkaloid, which is an agonist at neuronal nicotinic acetylcholine receptors [37,38].

In this study, extensive sampling was performed throughout Spain, along an environmental gradient that focused especially on benthic aquatic systems, including aerophytic and endolithic habitats, in an attempt to broaden the perception of the environmental presence of cyanotoxins.

## 2. Results

**Figure 2:**
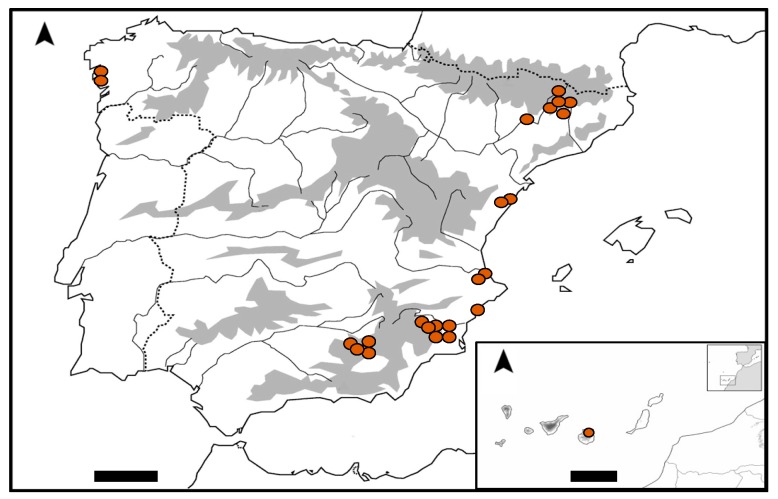
Map of the study area with sampled localities. Grey areas indicate mountain systems whose altitude is higher than 1500 m above sea level (asl). The insert represents the Canary Islands. Scale bars represent 100 km.

Different habitats were sampled throughout Spain, from both Atlantic and Mediterranean regions. These habitats were high mountain streams or creeks of the Pyrenees and Sierra Nevada mountains, peat bogs from the Pyrenees, middle mountain streams from northeast and southeast Spain and the Canary Islands, saline and freshwater lagoons from northwest Spain, saline and freshwater springs from Mediterranean marshes, and a cave and building located in the southeast (Figure 2, Table 1).

**Figure 3:**
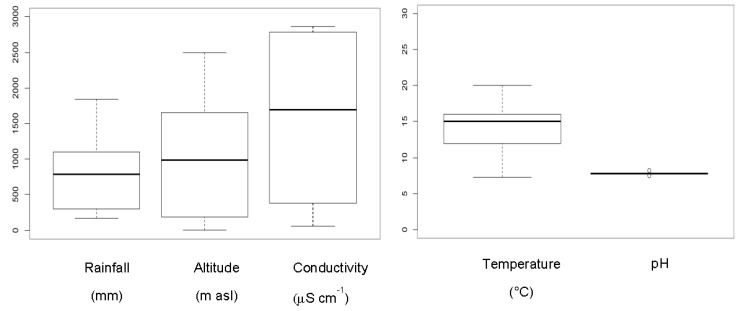
Ranges of the main ecological variables in the sampled habitats.

Most areas were granitic, but others were calcareous. Most of the samples contained freshwater, but some had saline water. The range of altitudes varied from 5 m to 2500 m above sea level. Rainfall ranged between <200 mm and 1843 mm. The mean air temperature was between 5 and 22.5 °C. Conductivity varied between 61.6 and 2850 μScm^−1^, with pH between 7 and 8.5 (Figure 3).

**Figure 4:**
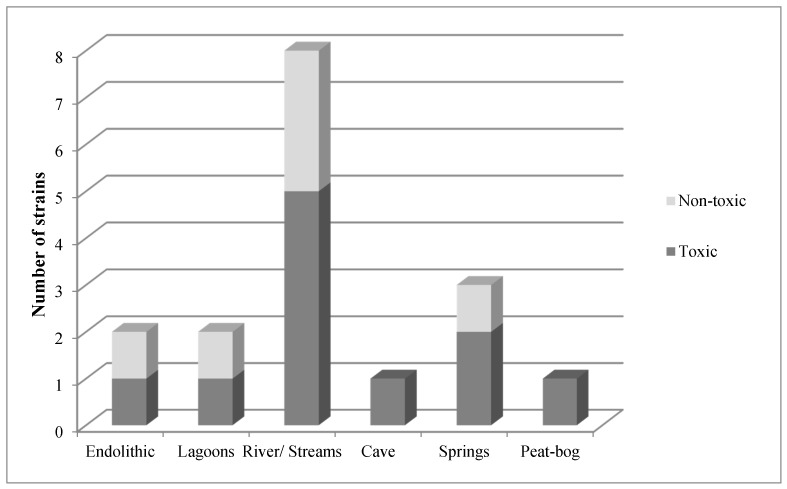
Distribution of the toxic strains in each habitat.

Toxic species were detected in all the sampled habitats, including both aerophytic and endolithic species (Figure 4). The proportion of toxic strains varied between 28% and 100% of all the strains studied. Table 2 below lists all the isolated and extracted species.

**Figure 5:**
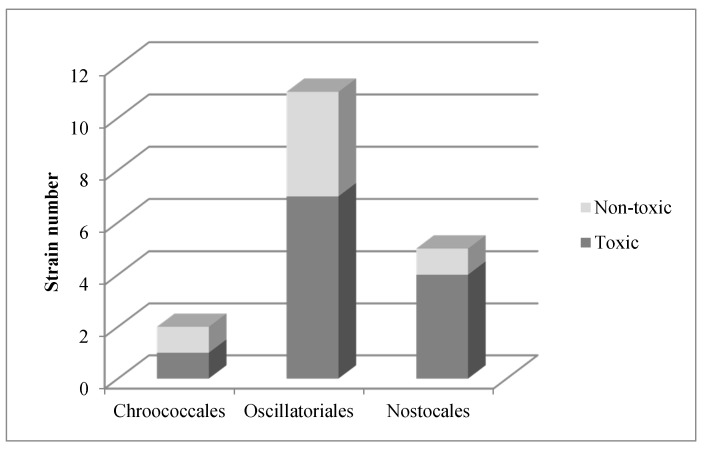
Distribution of the toxic strains in currently recognised taxonomic orders.

Eleven of the 24 analysed strains contained microcystin. The proportion of toxic strains to non-toxic strains was similar for different taxonomic orders of cyanobacteria, and varied between 50% and 70%: 50% in Chroococcales, 60% in Oscillatoriales and 67% in Nostocales (Figure 5).

**Figure 6:**
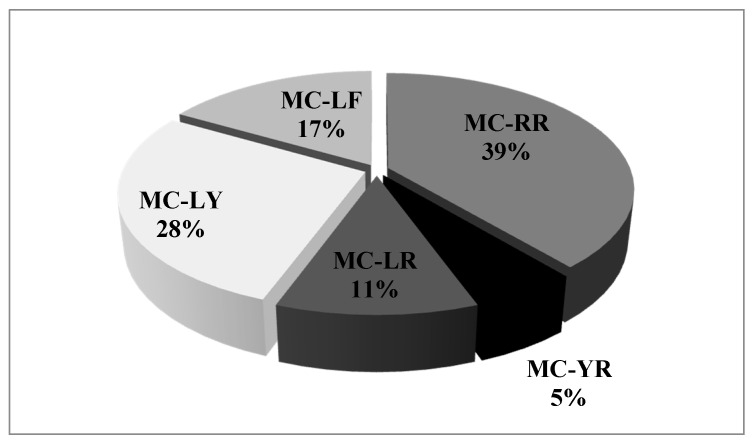
Frequency of each of the detected microcystin variants.

The number of microcystin variants detected per strain varied between one and three (Table 3). MC-RR was the most common microcystin in all of the studied strains (39%), followed by MC-LY (28%) and MC-LF (17%).The least common microcystins were MC-LR (11%) and MC-YR (5%) (Figure 6).

**Figure 7:**
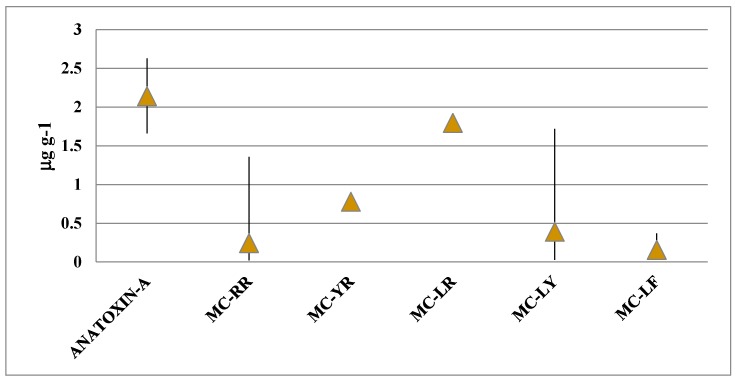
Mean and range of the concentrations of each of the identified variants.

MC-LR reached the highest concentration per dry weight (1.87 μg/g), followed by MC-LY (1.72 μg/g), while MC-RR (1.36 μg/g), MC-YR (0.78 μg/g), and MC-LF (0.37 μg/g) were less concentrated. Anatoxin-a reached the highest concentration of all the toxins (2.63 μg/g), and was present in three strains from Oscillatoriales (Table 3, Figure 7).

## 3. Discussion

The ability to synthesise microcystins appeared very early in the evolution of cyanobacteria [39], but has been lost in certain phylogenetic branches over time. Most toxin screening efforts have been made in freshwater aquatic habitats, mainly lagoons and reservoirs. Running water, salt water, high mountains, peat bogs and aerophytic habitats (except for lichens) have been very rarely sampled [9,40].

**Figure 8:**
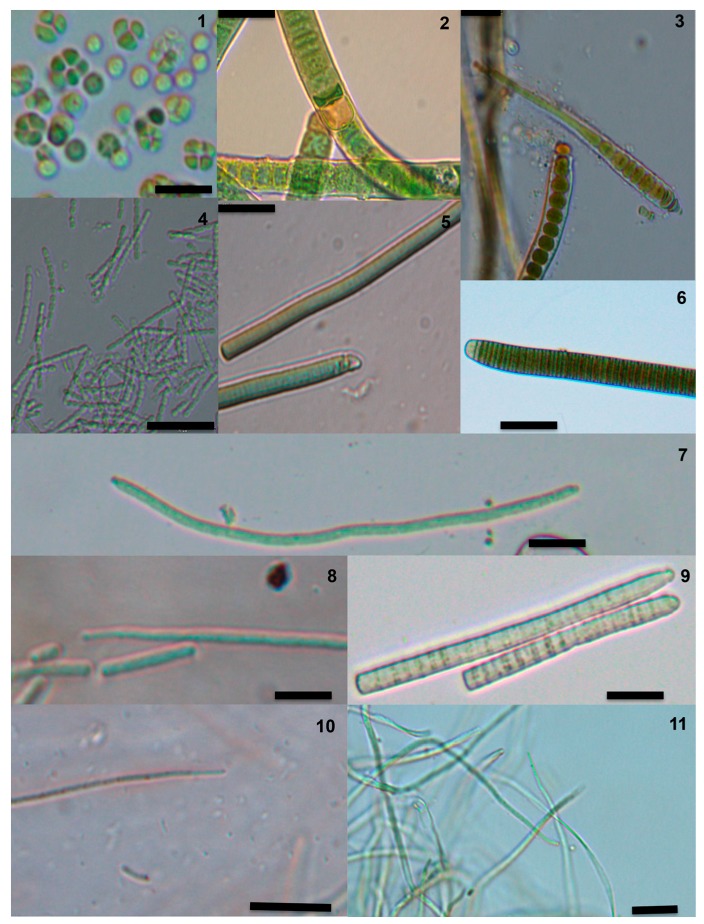
Images of the toxic strains that were isolated during this study: 1. *Pseudocapsa dubia*. 2. *Scytonema drilosiphon*. 3. *Gloeotrichia natans*. 4. *Pseudanabaena frigida*. 5. *Phormidium uncinatum*. 6. *Oscillatoria margaritifera*. 7. *Phormidium* sp. 8. *Geitlerinema splendidum*. 9. *Phormidium favosum*. 10. *Geitlerinema carotinum*. 11. *Leptolyngbya rivularianum*. The scale bar represents 20 μm.

In this work, microcystins were detected for the first time in seven species, found in high mountain streams, caves and endolithic habitats: *Pseudocapsa dubia*, *Gloeotrichia natans*, *Scytonema drilosiphon*, *Geitlerinema carotinum*, *Oscillatoria margaritifera*, *Pseudanabaena frigida*, and *Schizothrix rivularianum* (Figure 8). Anatoxin-a production was confirmed in *Phormidium uncinatum*, and was detected for the first time in *Geitlerinema carotinum* and *G. splendidum*.

The focus on freshwater environments is justified by the potential human health risks that the presence of microcystins may present for local populations [41]. However, since neither the factors that trigger the synthesis of these compounds nor their ecological function are known, significant further research must be conducted in all habitats and phylogenetic branches [17,42].

During the present study, we detected toxic strains in all of the studied habitats, which implies the need for more extensive reviews of the data from both the taxonomic and ecologic points of view. Our data showed that the frequency of microcystin producers was higher in Nostocales, although we found a similar proportion in Chroococales and Oscillatoriales. These data are similar to those found in other studies published elsewhere [15].

In most strains, more than one microcystin congener was detected. Interestingly, some of the most abundant congeners were those not considered to be particularly common. In particular, MC-LF and MC-LY were detected fairly frequently, and at relatively high concentrations. Even though these supposedly rare variants have probably always been present, they have only recently been widely reported [10,27] perhaps due to improved extraction and detection methods. These cogeners should be included in monitoring programmes, especially taking into account that the toxic capacity of the more hydrophobic variants such as MC-LF and MC-LY is sometimes much higher than MC-LR [22], which is typically used for risks assessments. The use of solid-phase extraction (SPE), coupled with immunoaffinity columns (IACs), improves the efficiency of extraction and clean-up of microcystins, especially in complex matrices [15]. The effects of exposure to MC-LF and MC-LW on the degradation, growth and proliferation of human cells is much higher than on the cells exposed to MC-LR, but phosphatase inhibition is much lower [22]. The toxicity of microcystin variants is strongly associated with the hydrophobicity of their structural amino acids. MC-LF and MC-LW therefore induce more pronounced cytotoxic effects on Caco-2 cells compared with MC-LR, and are taken up much more rapidly, or with a higher affinity, by human embryonic kidney cells and human primary hepatocytes [43,44].

Interest in algae as a food supplement has considerably increased of late. A new market has opened up for these products, which has the potential for vast economic benefits. Aquaculture has also undergone similar or even stronger growth, with the global yearly production of thousands of tons of fish and shellfish. However, no clear regulation or legislation exists for these products in most countries. Several authors have reported the presence of microcystins in food supplement products [45], as well as in fishponds and fish [26], but very little information and very few studied effects on consumer health are known. Increasing efforts to improve research in this field are needed by companies that prepare these products, not to mention by environmental agencies, countries, and governments, in order to create international regulations for blue-green algae supplements (BGAS), given their strong potential impact on populations’ health. Above all, international regulation is necessary, because BGAS is now available and easily sold on the internet, which increases possible risks worldwide.

Different types of quantification and detection kits are available, but the effectiveness for these “rare” variants is very low in some cases [38]. The use of several complementary identification or quantification methods should be compulsory, and the utilisation of biological tests based on phosphatase inhibition as the first step should be emphasised. These functional toxicity assays permit the detection of toxicants that may escape standard analytical detection [46,47,48]. Ward et al. [18] have already suggested that most strains usually produce more than one variant, and that the concentrations of some variants might drop below the level of detection for chemical methods, and could thus escape analytical control. Puddick et al. [49] provides an assessment of the microcystin congener diversity produced amongst cyanobacterial strains.

Climate warming will probably increase problems with cyanotoxins [42,43]. Thus, much work has to be done, in order to know how to prevent toxic events, how to avoid the conditions that promote their synthesis, and how to control cyanobacteria populations [50]. This is particularly important, as more hydrophobic variants will increase their predominance, given the current and future predicted atmospheric and environmental conditions [50,51,52].

Our data show that microcystins are widespread, in a variety of continental habitats, including high mountain streams, peat bogs, and aerophytic and endolithic communities. The results from our study demonstrate how important it is that screening for variants be intensified and globally managed, in order to prevent sanitary and environmental problems. Cyanotoxin-monitoring programmes worldwide should be revised to include a wider range of toxin variants (as has been done in some countries), or to initially test toxins according to “in vitro” toxicity assays, such as protein phosphatase inhibition.

## 4. Materials and Methods

### 4.1. Study Area

Extensive sampling was performed throughout Spain (including the Canary Islands), from high mountain streams, such as those in the Pyrenees and Sierra Nevada mountains, medium and low mountain streams in the southeast, freshwater and saltwater springs from Mediterranean marshes, lagoons within Atlantic marshes, caves, and buildings. Most strains lived epilithically, but some were epipelic, and others were endolithic (chasmo or euendolithic) (Table 1).

### 4.2. Culturing

All the collected samples were isolated in different culture media (Bold’s Basal Medium with soil extract, BG-11 for freshwater or aerophytic strains, and SWES for saltwater species) and maintained as monoclonal cultures in the Algology Laboratory at Murcia University. Strains were transferred to 500-mL flasks, and were maintained with BG-11 in aeration at 20 °C and 65–70 μM m^−2^ s^−1^ (PAR), in a 16:8 light-dark photoperiod, until enough biomass was obtained for the later analyses (25–1000 mg). The incubation period varied from 2 weeks to 2 months, depending on growth rates. Biomass was collected by filtration and was lyophilised, weighed, and kept frozen (−20 °C) until analysed [26]. The analysis was done 1 week after collection.

### 4.3. Taxonomic Identification

The monoclonal cultures were identified taxonomically by standard algological techniques under an OLYMPUS BX60 light microscope (OLYMPUS IBERIA S.A.U., Barcelona, Spain), equipped with a digital camera (OLYMPUS IBERIA S.A.U., Barcelona, Spain), following [53,54,55]. Comparisons were made with the preserved field material whenever necessary.

### 4.4. Microcystin Extraction and Quantification

Freeze-dried and weighed biomass was ground with ground-glass homogenizers. Microcystins were extracted by sonicating for 30 min with 75% methanol, following the protocols of [36,37,48,52,56]. Cycles were repeated three times. Extracts were kept frozen (−20 °C), and were then concentrated in a Büchi Vac^®^ V-500 vacuum dryer (BÜCHI Labortechnik AG, Postfach, Switzerland) and re-suspended in methanol for three cycles. Dried extracts were re-suspended in 1 mL of HPLC grade methanol, transferred to a 1.5-mL vial, and centrifuged in a Spectrafuge 24D microcentrifuge by Labnet International Inc. (Edison, New York, NY, USA) at 16 g for 5 min [26]. The resultant extracts were filtered through a regenerated 0.45 μm cellulose filter (Filter-Lab^®^, Eaton Filtration LLC, Tinton Falls, NJ, USA) and kept frozen until analysed [26]. Extracts were collected with a 4¾ inch-long hypodermic needle (B/Braun Sterican^®^, B Braun España, Barcelona, Spain) attached to a 1-mL polypropylene syringe (BD Plastipak^TM^, BD, Madrid, Spain) and placed inside 1.8 amber phials (Scharlab S.L., Barcelona, Spain).

The identification and quantification of microcystins were performed by HPLC in a high-resolution photodiode array detection analysis, using a HPLC-VWR Hitachi equipped with an L-2130 Diode Array model L-2455, following the recommendations of [27,45,46,48]. Analytes were separated in a reverse-phase Agilent silicon Zorbax column C18 (4.6 × 250 mm × 5µm). The gradient mobile phase was composed of water and acetonitrile (ACN), and was acidified with trifluoroacetic acid (TFA) (0.5 μL L^−1^). The flow rate was 1 mL min^−1^ and the injection volume was 20 μL. The results were confirmed by HPLC/MS TOF (Agilent 6220, Agilent Technologies Spain S.L, Madrid, Spain).

The Anatoxin-a was extracted following the recommendations of [35,36], and was identified and quantified by HPLC photodiode array detection (Hitachi, Barcelona, Spain) and HPLC/MS (Agilent Technologies Spain S.L, Madrid, Spain).

### 4.5. Chemicals

All the reagents were HPLC-grade and purchased from Sigma-Aldrich (St. Louis, MO, USA). The ultrapure grade water (Milli-Q water) came from the Millipore Corporate (Bedford, MA, USA). The standards of microcystins (MC-LF, MC-LR, MC-LY, MC-RR, MC-YR) and Anatoxin-a were obtained from Enzo Life Sciences (Farmingdale, NY, USA).

## Figures and Tables

**Table 1:** List of the sampled localities, indicating habitat type, lithology, altitudes, geographic coordinates, mean rainfall, and mean temperature.

Localities	Habitat	Lithology	Altitude(m)	Coordinates	Rainfall(mm)	Mean Temperature (°C)
Prado Redondo, Sierra Nevada, Granada	Creek	Granitic	2100	37°05′23.0″ N 3°24′56.8″ W	1322	7.8
San Juan, Sierra Nevada, Granada	Creek	Granitic	2500	37°05′16.7″ N 3°22′18.5″ W	1322	7.8
Poqueira, Sierra Nevada, Granada	Creek	Granitic	1540	36°59′26.8″ N 3°21′00.2″ W	935	11.6
Vall de Mulleres, Vielha, Lleida	Peat bog	Granitic	1609	42°37′40.6″ N 0°45′34.9″ E	1843	8.5
Riu Escrita, Lleida	Stream	Granitic	1700	42°34′38″ N 0°56′52″ E	1100	5.0
Fonts Lac S. Maurici, Lleida	Springs	Granitic	1910	42°32′28.9″ N 9°01′21.6″ W	1100	5.0
Riu Llebreta, Lleida	Stream	Granitic	1999	42°34′38″ N 0°56′52″ E	1100	5.0
Riu Ter, Villalonga, Girona	River	Granitic	1067	42°19′59″ N 2°18′47″ E	933	9.8
Lagoa Carregal, Corrubedo, A Coruña	Lagoon	Granitic	5	42°33′002″ N 9°02′00″ W	933	9.8
Lagoa Vixán, Corrubedo, A Coruña	Lagoon	Granitic	5	42°33′002″N 9°02′00″W	1014	14.8
Guayadeque, Gran Canaria	Stream	Volcanic	1273	27°56′00.7″ N 15°28′57.7″ W	175	22.5
Palacio Guevara, Lorca, Murcia	Building	Marble	353	37°40′29.82″ N 1°41′51.54″ W	232	17.6
Río Alhárabe, Moratalla, Murcia	Stream	Calcareous	900	38°12′50″ N 1°57′46″ W	522	15.7
Azud de Ojós, Ojós, Murcia	Reservoir	Calcareous	132	38°8′ 52″ N 1°20′32″ W	306	17.4
Cueva de los Grajos, Cieza, Murcia	Cave	Calcareous	250	38°14′21″ N 1°25′08″ W	307	17.2
Río Chícamo, Abanilla, Murcia	Stream	Calcareous	290	38º24′97″ N 1º00′18″ W	<200	10.0
Marjal Almenara, Almenara, Castellón	Freshwater Spring	Calcareous	26	39°44′54.1″ N 0°11′17.3″ W	467	17.5
Marjal de Pego-Oliva, Valencia	Saline Spring	Calcareous	5	38° 52′08.2″ N 0°02′57.92″ W	637	17.8
Río Amadorio, Vilajoyosa, Alicante	Stream	Calcareous	32	38°30′19″ N 0°13′58″ W	300	18.0
Río Algar, Callosa, Alicante	Stream	Calcareous	247	38°39′33.67″ N 0°5′45.58″ W	519	16.9

**Table 2:** Isolated and extracted strains. Taxonomic order, locality and habitat are listed. Toxicity is highlighted in bold (dark grey).

Taxa	Order	Habitat	Locality
*Hyella balani* Lehman	Chroococcales	Euendolithic, spring	Marjal Oliva-Pego, Valencia
*Pseudocapsa dubia* Ercegovic	Chroococcales	Chasmoendolithic	Palacio Guevara, Lorca, Murcia
*Gloeotrichia natans* (Hedwig) Rabenhorst ex Bornet et Flahault	Nostocales	Epiphytic, lagoon	Lagoa Vixán, Corrubedo, A Coruña
*Nostochopsis lobata* Wood ex Bornet & Flahault	Nostocales	Epiphytic, lagoon	Lagoa Vixán, Corrubedo, A Coruña
*Rivularia biasolettiana* (Meneghini ex Bornet & Flahault	Nostocales	Epilithic, stream	Río Alhárabe, Moratalla, Murcia Río Chícamo, Abanilla, Murcia
*Scytonema drilosiphon* Elenkin & V. I. Polyansky	Nostocales	Epilithic, cave	Cueva Grajos, Cieza, Murcia
*Geitlerinema splendidum* (Greville ex Gomont) Anagnostidis	Oscillatoriales	Epilithic, spring	Ullal Almenara, Castellón
*Homoeothrix juliana* (Bornet & Flahault ex Gomont) Kirchner	Oscillatoriales	Epilithic, stream	Río Amadorio, Alicante
*Leptolyngbya subtilis* (West) Anagnostidis	Oscillatoriales	Epilithic, spring	Ullal Almenara, Castellón
*Leptolygnbya truncata* (Lemmermann) Anagnostidis & Komárek	Oscillatoriales	Epilithic, stream	Río Amadorio, Alicante
*Oscillatoria margaritifera* Kützing ex Gomont	Oscillatoriales	Epilithic, spring	Ullal Almenara, Castellón
*Oscillatoria sancta* Kützing ex Gomont	Oscillatoriales	Epilithic, stream	Río Ter, Vilallonga, Lérida
*Geitlerinema carotinum* (Geitler) Anagnostidis	Oscillatoriales	Epipelic, reservoir	Azud Ojós, Ojós, Murcia
*Phormidium autumnale* (Agardh) Trevisan ex Gomont	Oscillatoriales	Epilithic, stream	Río Alhárabe, Moratalla, Murcia
*Pseudanabaena frigida* (Fritsch) Anagnostidis	Oscillatoriales	Epipelic, peat bog	Vall de Molleres, Viella, Lérida
*Schizothrix rivularianum* Voronichin	Oscillatoriales	Epipelic, stream	Río Alhárabe, Moratalla, Murcia
*Phormidium uncinatum* Gomont ex Gomont	Oscillatoriales	Epipelic, stream	Barranco Guayadeque, Gran Canaria

**Table 3:** Types of toxins present in the different isolated strains (nd = not detected).

Taxa	Locality	Microcystins	Anatoxins
*Gloeotrichia natans*	Corrubedo, Galicia	MC-LF, MC-RR	nd
*Geitlerinema carotinum*	Ojós, Murcia	MC-LY	ANT-a
*Geitlerinema splendidum*	Almenara, Valencia	MC-LF, MC-RR	ANT-a
*Nostoc* cf. *commune*	Sierra Nevada, Granada	MC-LF, MC-LY	nd
*Oscillatoria margaritifera*	Almenara, Valencia	MC-LF, MC-LY, MC-RR	nd
*Phormidium autumnale*	Moratalla, Murcia	MC-LR	nd
*Phormidium uncinatum*	Guayadeque, Gran Canaria	MC-LF, MC-LY	ANT-a
*Phormidium sp.*	Sierra Nevada, Granada	MC-LF, MC-LY	nd
*Pseudoanabaena frigida*	Vall de Mulleres, Lérida	MC-RR	nd
*Pseudocapsa dubia*	Palacio de Guevara, Murcia	MC-RR, MC-YR	nd
*Rivularia biasolettiana*	Río Chícamo, Murcia	MC-LR, MC-LY, MC-RR	nd
*Schizothrix rivularianum*	Río Alhárabe, Murcia	MC-LY, MC-RR	nd
*Scytonema drilosiphon*	Cueva de los Grajos, Murcia	MC-LY	nd
